# A comparison of visual and acoustic mismatch negativity as potential biomarkers in schizophrenia

**DOI:** 10.1038/s41598-023-49983-5

**Published:** 2024-01-10

**Authors:** Hajnalka Molnár, Csilla Marosi, Melinda Becske, Emese Békési, Kinga Farkas, Gábor Stefanics, István Czigler, Gábor Csukly

**Affiliations:** 1 Department of Psychiatry and Psychotherapy, Semmelweis University, Budapest, Hungary; 2 Institute of Cognitive Neuroscience and Psychology, RCNS, HU-RES, Budapest, Hungary

**Keywords:** Biomarkers, Preclinical research, Neuroscience, Schizophrenia

## Abstract

Mismatch negativity (MMN) is an event-related potential (ERP) component generated when an unexpected deviant stimulus occurs in a pattern of standard stimuli. Several studies showed that the MMN response to both auditory and visual stimuli is attenuated in schizophrenia. While previous studies investigated auditory and visual MMN in different cohorts, here we examined the potential clinical utility of MMN responses to auditory and visual stimuli within the same group of patients. Altogether 39 patients with schizophrenia and 39 healthy controls matched in age, gender, and education were enrolled. We recorded EEG using 64 channels in eight experimental blocks where we presented auditory and visual stimulus sequences. Mismatch responses were obtained by subtracting responses to standard from the physically identical deviant stimuli. We found a significant MMN response to the acoustic stimuli in the control group, whereas no significant mismatch response was observed in the patient group. The group difference was significant for the acoustic stimuli. The 12 vane windmill pattern evoked a significant MMN response in the early time window in the control group but not in the patient group. The 6 vane windmill pattern evoked MMN only in the patient group. However, we found no significant difference between the groups. Furthermore, we found no correlation between the clinical variables and the MMN amplitudes. Our results suggest that predictive processes underlying mismatch generation in patients with schizophrenia may be more affected in the acoustic compared to the visual domain. Acoustic MMN tends to be a more promising biomarker in schizophrenia.

## Introduction

Schizophrenia is a severe mental disorder, with a lifetime prevalence of 1%^1^. The pathomechanism is still unclear, the diagnosis is based on a clinical interview. We are still lacking a biomarker, that may help with setting up the diagnosis, and with the follow-up of the pharmacological treatment or the prevention of relapses.

Mismatch negativity (MMN) is a widely investigated event-related potential (ERP) component, elicited in sequences of visual or acoustic stimuli (standards), interspersed by infrequent (deviant) stimuli (oddball paradigm)^2,3^. MMN is elicited automatically, i.e., by unattended^4^ and task-irrelevant stimuli^5^. The onset of auditory MMN (aMMN) is observed from 50 ms and with a peak between 100 and 250 ms post-stimulus. Latency of visual mismatch negativity (vMMN) is usually longer. However, in both modalities the latency and amplitude varies according to the stimulus characteristics^6^.

MMN is studied in several psychiatric disorders. Umbricht and colleagues described that patients with major depression or bipolar disorder did not show significantly decreased MMN signal compared to matched control subjects^7^. Auditory MMN is impaired in bipolar disorder with psychotic features, but in a lesser extent, compared to schizophrenia. Patients with ADHD do not show significant MMN impairment, compared to healthy control subjects^8^. These findings indicate that MMN impairment tends to be somewhat specific to schizophrenia. Because of the easy, inexpensive and quick measurement and the robust results in schizophrenia, mismatch negativity is considered as a potential biomarker of the disorder^9^. Compared to other biomarker candidates (P50, N100, P300), MMN showed the greatest effect size^10^. Patients with reduced mismatch negativity amplitude showed a significantly worse performance on the tests of working memory, emotion recognition^11^ and attention/information processing^12^, which indicates that MMN detection is closely related to cognitive impairments in schizophrenia^13,14^, or is even connected to cognitive dysfunction in general^15^. In the last years, MMN was intensely studied in different psychiatric disorders with cognitive decline. The result show different MMN latency or amplitude in patients with cognitive impairment, such as chronic alcoholism, multiple sclerosis or Alzheimer’s disease^16^, which indicates that MMN could be a useful tool to understand the pathophysiology of these psychiatric disorders^6^. MMN impairment worsens over the course of chronic schizophrenia. These findings suggest that MMN could be useful in monitoring the progression of schizophrenia^17^.Acoustic MMN can be examined with duration^18^, pitch^19^, frequency or complex deviants. Deficits in acoustic mismatch negativity (aMMN) generation in schizophrenia were first reported by Shelley and colleagues^20^ in 1991, who used duration-deviant stimuli. Duration-deviants are candidates to be a biomarker in schizophrenia, while pitch and frequency deviants showed a smaller effect size (see the meta-analysis^21^, or the review^22^). Bodatsch and colleagues^23^ found reduced MMN in at-risk subjects converting to first psychosis but not in nonconverters. Furthermore, Jessen and colleagues^24^ described reduced MMN in first-degree relatives of patients with schizophrenia compared to controls. These findings indicate that MMN examinations might help the prediction of conversion and prevention.

Visual mismatch negativity (vMMN) can be elicited by a high variability of different stimuli such as brightness changes^25^, facial gender difference^26^, facial emotions^27,28^ or even simple visual stimuli^29^. Compared to aMMN, there are relatively few clinical trials investigating vMMN in schizophrenia^2,30^. Farkas and colleagues found significantly decreased MMN in patients with schizophrenia, using simple visual stimuli^29^. Urban and colleagues used a motion-direction paradigm to compare the mismatch (MM) signal between schizophrenia patients and control subjects. Their results showed significantly decreased MMN in the patient group^31^.

Functional interpretation of auditory and visual MMN is connected to the predictive coding theory^4,6,32^. According to this theory, the brain creates a model to interpret the sensory input and this model serves to predict the following sensory input. Based on this model, the brain calculates the difference between the prediction and the real input, and updates the model based on the prediction error. It can be hypothesized that in pathological conditions alterations in this aspect generates a maladaptive model and it can lead to hallucinations and delusion. Patients with auditory hallucinations rely more on predictions than on sensory evidence, which leads to false assumptions and psychosis^33^. Patients with chronic schizophrenia have rigid response pattern. While patientsc with acute unmedicated psychosis show increased tendency to update their model according to the prediction error^34^.

Previous pharmaceutical studies found that NMDA-receptor antagonists, such as ketamine can trigger positive, negative and cognitive symptoms of schizophrenia^35^. These agents also caused the decrease of the auditory MMN signal in healthy volunteers, proving the link to NMDA-receptor function^36–38^. These findings led to the glutamate hypothesis of schizophrenia, which is supported by indirect and direct evidence. Postmortem studies found decreased NMDA-receptor density in the brains of patients with schizophrenia^39^. According to a previous meta-analysis NMDA-modulatory drugs caused significant improvement of positive and negative symptoms in the patients when added to their treatment^40^. The connection between vMMN and the glutamatergic system is not as well studied as the molecular background of aMMN, however Heekeren and colleagues observed vMMN reduction after the admission of ketamine, implying the involvement of the glutamatergic system in vMMN generation^38^.

The α7 nicotinic cholinoceptor function and nicotine intake also influence the MMN in schizophrenia. Nicotine increases the patients’ duration MMN amplitude to a level comparable with that seen in control subjects^41^. Furthermore, nicotine improves sensory gating, which has positive effect on negative symptoms. The smoking rate among schizophrenia patients is higher (90%) compared to the healthy population (20–30%). This behavior can be considered as “self-medication”, because nicotine improves sensory gating, thus has a positive effect on working memory and attention deficit^41^. Alpha 7 nicotinic receptor agonists found to have beneficial effect in animal studies and are promising drugs to treat the cognitive and negative symptoms in schizophrenia^42^. The connection to the MMN can help to monitor the effect of these drugs and to find new pharmaceutical agents to treat negative symptoms of schizophrenia.

The first EEG and MEG studies located the source of auditory MMN as single dipole generators within the region of bilateral superior temporal gyri (STG) in the vicinity of the transverse (Heschl's) gyrus^43,44^. Shinn and colleagues described increased functional connectivity in this region in schizophrenia patients^45^. Salisbury and colleagues^46^ described that mismatch negativity is reduced in chronic but not in first-episode schizophrenia, which is correlated with the Heschl-gyrus gray matter volume. These results could be explained by the findings of Kasai and colleagues, who recorded progressive Heschl-gyrus volume reduction in schizophrenia patients^47,48^. Later, surface and intracranial EEG, PET and fMRI research helped us to understand MMN generators in detail. The results of these studies also suggest the involvement of the inferior frontal gyrus, medial frontal gyrus and anterior cingulate cortex^49^. Li and colleagues described two pathways of MMN generation: first from the auditory cortex the signal propagates to the motor areas and then from the auditory cortex to the inferior frontal gyrus^50^. MEG studies located visual MMN in the occipital lobe^51^. Previous studies recorded structural, functional and metabolic changes in the occipital lobe of patients with schizophrenia^52^. Previous fMRI results described the involvement of the left inferior and middle frontal gyri in vMMN generation^53^. Most recent theories describe, that the visual mismatch response is modulated by a fronto-occipital network^54^.

In summary, the generation of aMMN and vMMN is strongly connected to the primary sensory cortices,and they share a partially overlapping network in the frontal cortex , which leads us to the assumption that there might be a correlation between the two modalities, however no previous study examined MMN generation in schizophrenia in both modalities.

Based on the above results, we hypothesize that patients with schizophrenia would show decreased auditory MMN over the central and frontal and decreased visual MMN at the occipital and frontal regions compared to neurotypicals. In addition, we expect a negative effect of symptom severity and antipsychotic dose and a positive effect of nicotine intake on MMN amplitudes. An exploratory part of the study was to investigate if auditory or visual MMN is more sensitive to diagnosis.

## Methods and materials

### Ethical statement

The study was approved by the Scientific and Research Ethics Committee of the Medical Research Council, Budapest, Hungary, and participants gave their written informed consent before the procedures. The experiments were carried out in full compliance with the Helsinki Declaration.

### Subjects

Altogether 39 patients with schizophrenia and 39 healthy control subjects matched in age, gender and education were enrolled to the study. Their demographic data is provided in Table 1. Selection criteria were the absence in their history of any central nervous system disorder, mental retardation, epileptic seizure and no history of head injury with loss of consciousness more than 10 min, and in addition at healthy participants, no history of psychiatric disease. All patients met the criteria for schizophrenia based on the Structural Clinical Interview for DSM IV (Diagnostic and Statistical Manual of Mental Disorders, Fourth Edition)^55^. Positive and Negative Syndrome Scale (PANSS) was evaluated by a trained psychiatrist^56^.

**Table 1:** Demographic information of both study groups and clinical characteristics of the schizophrenia group Mean (SD).

	Patient group	Control group
Gender(male/female)	27/12	27/12
Age	33.6 (10.5)	32.7 (9.7)
Education (years)	14.7 (2.9)	15.9 (2.1)
Handedness (left/right/ambidexter)	33/4/2	32/3/3
Illness duration	8.2 (8.4)	–
CPZ equivalent dose	267.4 (197.1)	–
PANSS total score	62.7 (17.6)	–

The abbreviations: CPZ, chlorpromazine equivalent dose; PANSS, Positive and Negative Syndrome Scale).

### Stimuli and procedures

Both visual and acoustic stimuli were used during the experiment. The visual stimuli consisted of 6 and 12 vane windmill patterns, the same as File and colleagues used in their previous study^57^. The stimuli were presented with Matlab2014a and it was in the middle of the monitor. The diameter of the windmill patterns was 32.28 angular degree, their contrast was high, 37.21 cd/m2 for bright and 0.14 cd/m2 for the dark segments. The colors were defined with RGB codes, as the following: the background was [0.3 0.3 0.3], the lighter parts of the windmill patterns were described as [0.6 0.6 0.6] and the darker parts were defined with the code [0 0 0 ]. The visual stimuli were visible for 200 ms and they were followed by a 600 ms long interstimulus interval. The presented acoustic stimuli were 100 ms and 200 ms duration binaural tones with 1000 Hz frequency. The volume of the tones was set according the hearing threshold of the subjests, which was measured before the examination.

**Figure 1:**
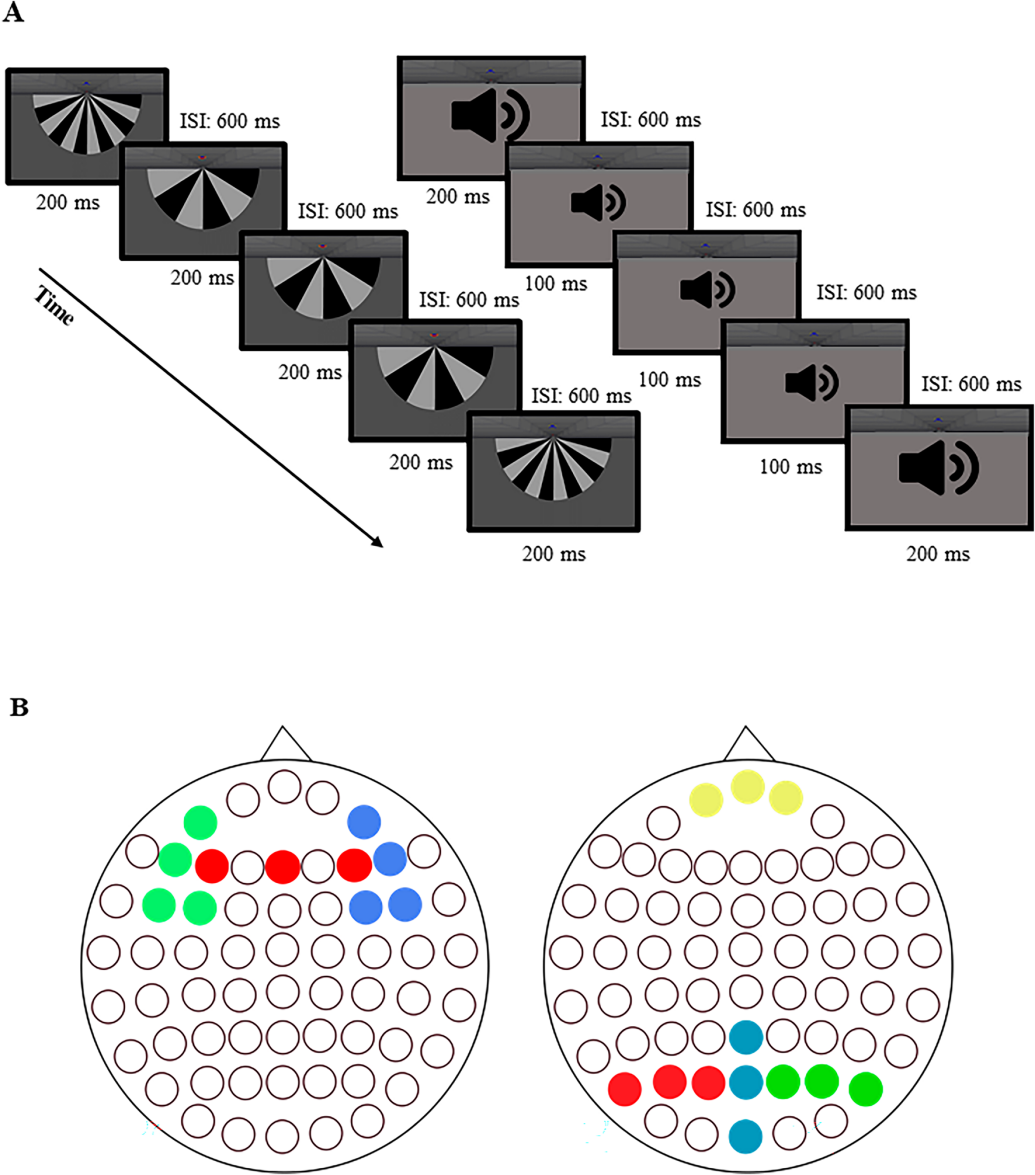
(**a**) Schematic illustration of the experimental paradigm. In the visual session, an oddball sequence of windmill patterns was presented for 200 ms followed by an inter-stimulus interval (ISI) of 600 ms. In the acoustic session, 100 and 200 ms long beeping sounds were presented, the stimuli were followed by an ISI of 600 ms. (**b**) The left scalp figure presents the acoustic regions of interest (green: left frontal (AF3, F5, FC5, FC3), red: frontocentral (F3, FZ, F4), blue: right frontal (AF4, F6, FC4, FC6)) the visual regions are shown in the right scalp figure (yellow:frontal(FP1, FPZ, FP2), red:left occipital (PO7, PO5, PO3), blue: midline occipital (PZ, POZ, OZ), green: right occipital (PO4, PO6, PO8)).

The experiment consisted of eight blocks. Each block contained a visual and an acoustic sequence, separated with a 15 s break. In each sequence 24 deviant and 125 standard, altogether 149 stimuli were presented. Altogether, during the EEG registration, 1000 visual standard and 192 visual deviant (sum of the 6 and 12 vane windmill patterns), 1000 acoustic standard and 192 acoustic deviant stimuli (sum of long and short acoustic stimuli) were presented. Deviant stimuli were distributed such that between each deviant stimuli we placed multiple standard stimuli. The number of standard stimuli between each deviant was randomly selected from the set {3,4,5,6,7}. We changed the deviant and standard stimulus in every session, so every visual and acoustic stimulus appeared four times as a deviant and four times as a standard durind the examination. This way we could remove adaptional bias. The schematic illustration of the paradigm is presented in Fig. 1. To maintain the subjects’ attention, a simple visual task was used. The subjects had to navigate a blue spaceship with the keyboard. They had to avoid the appearing red spaceships and catch the green spaceships. For more details, we refer to the article of Sulykos and colleagues^58^.

### EEG recording and processing

EEG was recorded with a 64 channel quikcap and a Curry7XS EEG recording system. The sampling rate was 1000 Hz. For data processing, the EEGLAB toolbox of Matlab^59^ was used. During the preprocessing, electrodes with many artifacts caused by movement were collected and interpolated with the mean of the surrounding electrodes. A 0.3 Hz highpass and a 40 Hz lowpass filter were applied on the data. Artifacts caused by movement or speech were eliminated by the ADJUST plugin, based on Independent Component Analysis (ICA). The final data was baseline corrected and rereferred to the average potential. EEG segments under − 80 µV and above 80 µV were rejected. EEG epochs of 600 ms with 100 ms prestimulus interval were selected for further analysis. At the end of the data processing, in average 169 visual deviant (SD = 26), 877 visual standard (SD = 133), 167 acoustic deviant (SD = 26), 866 acoustic standard (SD = 142) stimuli per subject were used for further analysis in the patient group. In the control group, in average 179 visual deviant (SD = 18), 929 visual standard (SD = 97), 198 acoustic deviant (SD = 19) and 928 acoustic standard (SD = 96) stimuli remained after the data processing.

### Data analysis

Mismatch negativity was calculated by subtracting *the response to the standard* from *the physically identical deviant*. We defined seven regions of interest (ROI) and the MMN amplitude was calculated as the mean amplitude inside these ROIs in the selected time window. The regions were selected according to previous studies^29,60–62^, the channels of the regions are presented on Fig. 1b. VMMN was examined in an early (160–200 ms) and a late (260–300 ms) time window, while we applied a single time window (120–200 ms) on the acoustic MMN epochs. The time windows were defined on previous study results^60,61,63^.

The effect of study group, ROI and their interaction on MMN amplitude was analyzed by a repeated measures mixed linear model (PROC MIXED in SAS 9.4) separately for acoustic and visual stimuli in the different time windows resulting in six different analyses altogether. Age and gender were included as covariates in all MIXED model analyses. Where the interaction of study group and ROI was significant post-hoc t comparisons were applied. Furthermore, we tested by post hoc t-tests in the mixed model if the MMN amplitude was different from zero in both study groups for all stimuli in all ROIs separately in order to detect if a mismatch signal was produced in the given group and ROI for a given stimulus. In case of post-hoc tests a Bonferroni correction for multiple comparisons were applied (level of significant p = 0.05/number of ROIs in the analysis).

Correlations between the clinical, demographical and the EEG data were analyzed by Pearson and Spearman correlations using R Studio and its packages. In the correlational analyses, also the Bonferroni correction for multiple comparisons was applied.

## Results

### Behavioral results

The control group was in average 77.89% successful in catching or avoiding spaceships (SD = 16.98). The patient group completed in average 69.91% of the task successfully (SD = 20.29).

### Acoustic MMN

#### Long acoustic

The results were analyzed in the 120–200 ms time window. In the control group, a significant MMN sign was detected in all three regions (left and right frontal and frontocentral), while no mismatch sign was detected in the patient group. Accordingly, the between group difference was significant (F(1;73) = 7.5, p = 0.008) indicating a decreased MMM in patients, while region (F(2;150) = 0.7, p = 0.51) and group * region (F(2;150) = 0.1, p = 0.86) effects were non-significant. The largest effect size was found in the right frontal region (Cohen’s d = 0.54).

#### Short acoustic

**Figure 2:**
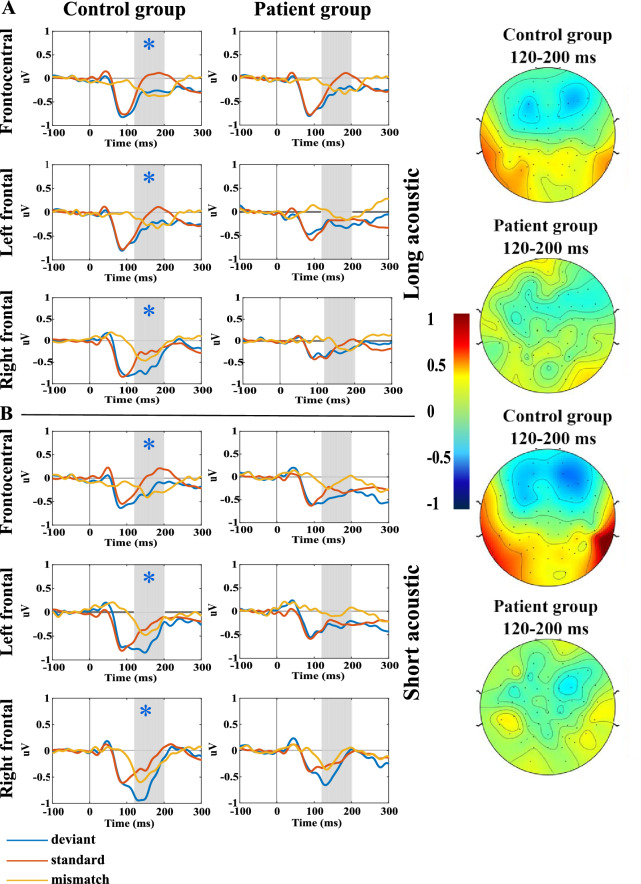
(**a**) Grand average ERPs and topoplots of the mismatch signal for long acoustic stimulus. The standard (red line) and the deviant stimuli (blue line), and the mismatch response (yellow line) from the three ROIs in the two study groups. The gray area shows the 120–200 ms time window. In the time windows marked with black * a significant mismatch signal was found. The blue star markes the significant different in the mismatch signal amplitude between the study groups. The topoplots show the mismatch responses in the 120–200 ms interval. (**b**) Grand average ERPs and topoplots of the mismatch signal for short acoustic stimulus.

The results were analyzed in the 120–200 ms time window and the results showed a similar pattern to the short stimulus type. In the control group a significant MMN was detected in all three regions (left and right frontal and frontocentral). The MMN signal in the patient group was not significantly different from zero, analysed by a post-hoc t-test. The between group difference was significant (F(1;73) = 13.5, p = 0.0005) indicating a decreased MMN in patients, while region (F(2;150) = 1.3, p = 0.27) and group * region (F(2;150) = 0.5, p = 0.58) did not have a significant effect. This stimulus had the largest effect size among all stimuli, in the left frontal region (Cohen’s d = 0.69). The topoplots and the line plots of auditory MMN are presented in Fig. 2.

### Visual MMN

#### V6 windmill stimulus

No mismatch negativity was found (corrected p > 0.05) in the early time window (160–200 ms) in any of the study groups. In the late time window (260–300 ms) a significant vMMN was detected in the frontal and right occipital regions in patients, while no vMMN was detected in the control group. However, neither between group difference was detected, nor the region * group interaction had a significant effect on the MMN amplitude. The effect of region was significant (F(3;228) = 4.6; p = 0.004). The effect sizes were in a wide range, the smallest was calculated in the midline occipital region in the early time window (Cohen’s d = 0.04), the largest was found in the frontal region in the same time window (Cohen’s d = 0.44).

#### V12 windmill

**Figure 3:**
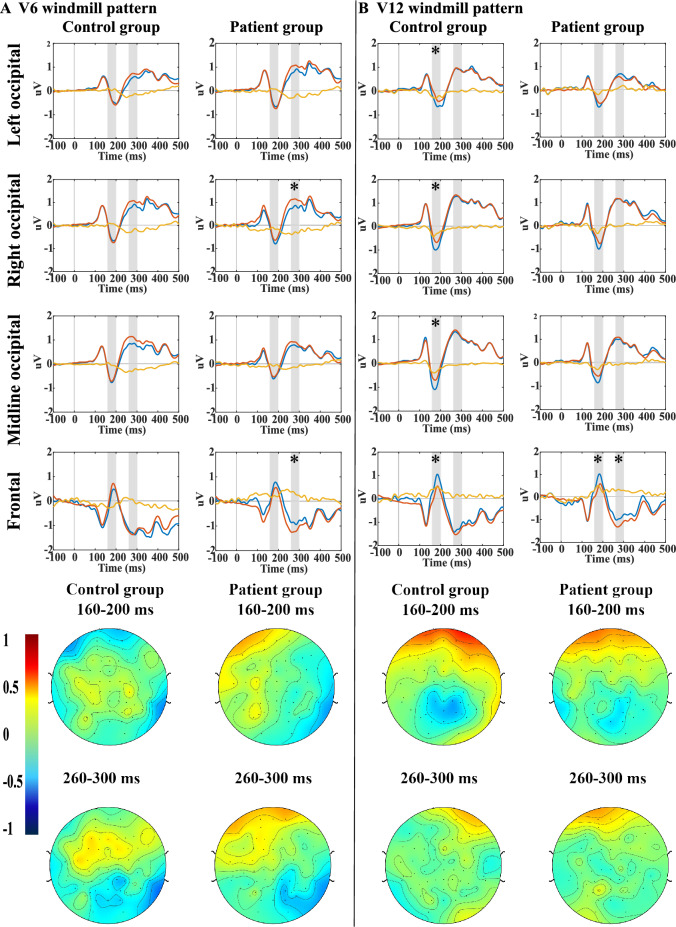
(**a**) Grand average ERPs and topoplots of the 6 vane windmill pattern as visual stimulus. (**b**) Grand average ERPs and topoplots of the 12 vane windmill pattern as visual stimulus.

VMMN was detected in the control group in all four regions (frontal, right occipital, left occipital and midline occipital) in the early time window (160–200 ms), while in the patient group a mismatch signal was only detected in the frontal region. No between group difference (F(1;73) = 2.3; p = 0.13) or group*ROI interaction (F(3;225) = 0.5, p = 0.69) was detected. The effect of region was significant (F(3;228) = 25.8; p < 0.0001). In the late time window (260–300 ms) significant MMN was detected in patients only in the frontal region, while no vMMN was detected in controls in any regions. There was no between group or inter-region difference or interaction effect. The right occipital region in the early time window showed the largest effect size (Cohen’s d = 0.26). The topoplots and the line plots of visual MMN are shown in Fig. 3.

### Correlation

No significant correlation (p ≤ 0.005) was found between the demographic variables and MMN amplitude. There was no significant correlation of MMN amplitudes with chlorpromazine equivalent antipsychotic dose (p = 0.038–0.904), illness duration (p = 0.101–0.746) or PANSS scores (PANSS total score: p = 0.04–0.728, PANSS positive subscale: p = 0.047–0991, PANSS general subscale p = 0.038–0.961, PANSS negative items p = 0.13–0.602). Furthermore, no significant correlation was found between the visual and acoustic MMN amplitude in any region (p = 0.085–0.928), analyzed by Pearson correlation. The patients’ nicotine intake (cigarettes/day) shows significant correlation with the amplitude of the auditory MM signal in the frontocentral region (p = 0.004, r = − 0.46) and in the right frontal region (p = 0.005, r = − 0.45), analyzed by Spearman correlation. The results describe no significant correlation with patients’ nicotine intake and vMMN in any region (p = 0.23–0.99, r = − 0.05 to 0.2).

## Discussion

This was the first investigation studying both acoustic and visual MMN on the same population of patients with schizophrenia and neurotypical control subjects.

Decreased auditory mismatch negativity was found in the patient group relative to controls independent of referencing (average or mastoid). This robust difference is consistent with previous findings^64,65^. While using the average reference no auditory mismach reponse was detected in the patient group, they showed a significant mismatch response after rereferencing to the average of mastoid electrodes, which produced a better signal-to-noise ratio (supplementary material). Auditory mismatch negativity (aMMN) is thought to reflect low level auditory processing. According to the predictive coding theory, the human brain tries to predict the subsequent stimulus based on the model representing previous regularity of information. An unexpected stimulus creates a prediction error, which modifies the model. In this theoretical framework, our finding, i.e., decreased auditory MMN can be interpreted as a signature of impaired predictive coding in schizophrenia. The reason of such impairment can be considered as the increased role of prediction, in comparison to the actual sensory input^34^.

Our results showed no significant correlation between aMMN and vMMN in any ROI, indicating that partially different brain networks are responsible for decreased acoustic and visual mismatch generation in schizophrenia. This lack of correlation could be explained by the pattern of different impairments for each person in the primary cortices responsible for acoustic and visual mismatch generations. The generators of aMMN are located in the primary acoustic sensory cortex. Previous fMRI studies described a difference in the Heschl gyrus volume and connectivity in schizophrenia, which can be the cause of the impaired acoustic stimulus processing and acoustic MMN^45,47^ in patients with schizophrenia. The vMMN is connected to the primary visual cortex in the occipital lobe, which also shows structural and functional impairments in schizophrenia^52^. Several previous studies shows the involvement of the frontal cortex in mismatch generation in both modalities^50,53,54^. The aMMN is found to be connected to the right prefrontal, inferior and middle frontal gyrus^66,67^, while the left inferior and middle frontal cortices take part in the generation of the vMMN^53^. A recent fMRI study by Grundei described an overlapping network , involving the inferior frontal cortex, the temporo-parietal areas and sensory cortices^68^. Future fMRI studies applying both vMMN and aMMN paradigms are needed to identify the common pathways of visual and acoustic mismatch generation and their impairment in schizophrenia and other neuropsychiatric disorders^16^. While there is strong evidence that NMDA function plays a crucial role in acoustic mismatch generation^36^, this link to visual mismatch generation is still unclear. Investigations administarating NMDA antagonist such as ketamine and applying vMMN paradimgs need to reveal this connection.

Though the results are controversial, glutamatergic transmission still remains a promising drug targets in schizophrenia, especially in terms of negative symptoms and cognitive deficit^69,70^. NMDA-receptor modulation can also be part of treatment of mood disorders or Alzheimer’s disease^71,72^. Our results show significant correlation between the acoustic MMN amplitude and the patients’ nicotine intake, indicating that nicotine is associated with the generation of MMN. This finding is in line with previous EEG studies showing that nicotine admission altered visual and acoustic mismatch generation compared to placebo^73,74^. Interestingly, these findings could not be reproduced by de la Salle et al.^75^, who did not find significant difference between the two study groups in the same setting. Therefore, it is still need to be clarified if the connection between the nicotinerg system and MMN generation is direct or is influenced by other social, enviromental or medical factors. Preclinical studies described that nicotine had beneficial effect on cognitive performance in schizophrenia patients^76^. This conclusion led to the intensive study of alpha 7 nicotinic receptor agonists as candidates for treatment of cognitive impairment^42^. AMMN could be helpful in monitoring the effect of these drugs.

Fisher et al.^77^ described a correlation between MMN amplitude and hallucinatory traits. In the present study we did not find correlation between the symptom severity and the MMN amplitude in the patient group. However, our results can be biased by the fact that we only examined patients with lower symptom severity and PANSS scores due to the need for compliance in a high density EEG experiment. We have to note here, that in our previous study^29^, we did not find correlations between MMN and symptom severity.

This is also the first experiment with schizophrenia patients using the windmill patterns in a vMMN paradigm. In control subjects, vMMN for the V12 windmill pattern was detected both in the frontal and occipital regions, while in patients vMMN appeared only over the frontal region, but between-group difference was not detected in visual stimuli in any regions. This pattern of result is consistent with previous studies using the same paradigm with healthy controls^78^. We detected vMMN in the patient group only in the late time window at the frontal region. This finding, taken together with the results of several previous studies (for a review see^2^) indicate that MM generation to visual stimuli also might be impaired in schizophrenia. Interestingly, the V6 windmill pattern produced a vMMN only in patients. A possible solution is suggested by File and colleagues, who obtained that V6 did not elicit vMMN in healthy participants, because this stimulus does not carry new information compared to the V12 pattern as the V12 stimulus contains the V6 pattern implicitly but not vice versa (V12 and V6 patterns were used as standard/deviant counterparts during the experiment). In other words, representation of the more complex stimulus included the representation of the less complex stimulus^57^. The frontal localization of the V6 mismatch negativity in patients suggest the involvement of a higher level processing mechanism, such as attention orientation and triggering^53^. Since we detect vMMN in both groups, it is unlikely that the lack of group difference is a result of a methodological issue in the design of our visual experiment. This view is also supported by the fact the same visual MMN paradigm was successfully applied in previous studies with similar negative results at V6 (but not V12) patterns in healthy participants.

Our results showed medium effect sizes (Cohens d = 0.46–0.69) to duration deviant auditory stimuli and small (non-significant) effect sizes to visual stimuli (Cohen’s d = 0.003–0.44). Our results are in line with previous meta-analyses describing the largest effect size (Cohen’s d = 0.79–0.88 (95% confidence interval)) for duration deviants compared to other acoustic deviant types^21^. The robust difference between the study groups suggests, that aMMN can be a more reliable biomarker for monitoring drug effects and drug development, than vMMN. Mismatch negativity is still a promising candidate, since MMN impairment thought to be selective to schizophrenia^8,79^. Previous studies found reduced MMN in at-risk subjects converting to first psychosis but not in nonconverters and even in first degree relatives of schizophrenia patients^23,24^. The impairment persists long term and tends to be correlated with disease progression. However, as Erickson and colleagues suggest this relationship with disease progression is not linear. The MMN impairment worsens within the first years after the diagnosis and stabilizes after the critical period has passed^79^.

## Limitations

Because of the long EEG experiment and the need for compliance, only patients with good functioning and compliance were enrolled. Therefore, patients with high symptom severity were excluded from the research. We only examined medicated patients, however no significant correlation was found between antipsychotic medication dose (in terms of CPZ equivalent) and the amplitudes of the MMN mismatch. The demanding visual task can be a limitation of the observation of the MMN.

## Conclusion

According to our results, acoustic mismatch generation tends to be impaired more in schizophrenia compared to visual mismatch generation. Therefore, aMMN is a more adequate candidate for biomarker in schizophrenia. Glutamatergic and nicotinic transmissions are still promising drug targets in schizophrenia, while MMN is connected to both NMDA and nicotinic function, which connection makes it a potential biomarker for monitoring the effect of drugs affecting these neurotransmitter systems.

### Supplementary Information

Supplementary Information.

## Data Availability

The datasets that are used and/or analyzed during the current study are available from the corresponding author on reasonable request.
