# Supplementary material for: A comparison of visual and acoustic mismatch negativity as potential biomarkers in schizophrenia
Source: Sci Rep. 2024 Jan 10;14:992. doi: 10.1038/s41598-023-49983-5 (PMC10782025; doi:10.1038/s41598-023-49983-5)
Supplement: Supplementary file 1 — Supplementary Information. [file 41598_2023_49983_MOESM1_ESM.docx]

Acoustic MMN references to the average of the mastoid electrodes

Long acoustic

The results were analyzed in the 120-200 ms time window. In the control group, a significant MMN sign was detected in all three regions (left and right frontal and frontocentral), while no mismatch signal was detected in the patient group (corrected p>0.05). Neither the between group difference (F(1;73)=0.6, p=0.43), nor the region (F(2;150)=0.99, p=0.37) and group * region (F(2;150)=0.05, p=0.95) effects were significant.

Short acoustic

The results were analyzed in the 120-200 ms time window. In the control group, a significant MMN was detected in all three regions (left and right frontal and frontocentral). The MMN signal in the patient group differed significantly from zero in the frontocentral and right frontal regions, analyzed by a post-hoc t-test. The between-group difference was significant (F(1;73)=6.9, p=0.01), indicating a decreased MMN in patients, while region (F(2;150)=1.7, p=0.19) and group * region (F(2;150)=0.5, p=0.62) did not have a significant effect. This stimulus had the largest effect size among all stimuli in the left frontal region (Cohen’s d=0.52). The line plots of auditory MMN are presented below.

Figure 1: Line plots of the MMN for the long acoustic stimulus. The star indicates the presence of the MMN in the time window.

Figure 2: Line plot of the short acoustic stimulus. In the regions marked with stars the MMN signal was significant. The blue star indicates a significant between-group difference
